# Status report on hypertension in Africa - Consultative review for the 6th Session of the African Union Conference of Ministers of Health on NCD's

**DOI:** 10.11604/pamj.2013.16.38.3100

**Published:** 2013-10-05

**Authors:** Steven van de Vijver, Hilda Akinyi, Samuel Oti, Ademola Olajide, Charles Agyemang, Isabella Aboderin, Catherine Kyobutungi

**Affiliations:** 1 APHRC, African Population and Health Research Center, Nairobi, Kenya; 2 AIGHD, Amsterdam Institute for Global Health and Development, The Netherlands; 3 Department of Health, Nutrition and Population, African Union Commission, Addis Ababa, Ethiopia; 4 Department of Public Health, Academic Medical Center, the Netherlands

**Keywords:** Hypertension, Africa, risk factor, treatment, control, cardiovascular diseases

## Abstract

Hypertension has always been regarded as a disease of affluence but this has changed drastically in the last two decades with average blood pressures now higher in Africa than in Europe and USA and the prevalence increasing among poor sections of society. We have conducted a literature search on PubMed on a broad range of topics regarding hypertension in Africa, including data collection from related documents from World Health Organization and other relevant organizations that are available in this field. We have shared the initial results and drafts with international specialists in the context of hypertension in Africa and incorporated their feedback. Hypertension is the number one risk factor for CVD in Africa. Consequently, cardiovascular disease (CVD) has taken over as number one cause of death in Africa and the total numbers will further increase in the next decades reflecting on the growing urbanization and related lifestyle changes. The new epidemic of hypertension and CVD is not only an important public health problem, but it will also have a big economic impact as a significant proportion of the productive population becomes chronically ill or die, leaving their families in poverty. It is essential to develop and share best practices for affordable and effective community-based programs in screening and treatment of hypertension. In order to prevent and control hypertension in the population, Africa needs policies developed and implemented through a multi-sectoral approach involving the Ministries of Health and other sectors including education, agriculture, transport, finance among others.

## Background

### Global Perspective

Hypertension, otherwise known as high blood pressure, is a leading cause of cardiovascular disease (CVD) worldwide [1]. The proportion of the global burden of disease attributable to hypertension has significantly increased from about 4.5 percent (nearly1 billion adults) in 2000 [2], to 7 percent in 2010 [3]. This makes hypertension the single most important cause of morbidity and mortality globally and highlights the urgent need of action to address the problem [4].

### Hypertension in Low- and Middle-income Countries

Until recently, hypertension was mainly associated with more affluent regions of the world. However, the condition is increasingly emerging in low and middle-income countries (LMICs) [5, 6] where health resources are scarce and stretched by a high burden of infectious diseases such as HIV, malaria and tuberculosis, and where awareness and treatment levels on hypertension control are still very low [6]. Currently, the worldwide burden of hypertension is greatest in LMICs where it affects about 1 in every 5 of the adult population and this is projected to increase [7]. By 2025, almost 3 out of every 4 people with hypertension will be living in LMICs. The absolute numbers affected by hypertension in LMICs are therefore considerably higher and are likely to increase as globalization and economic advancement usher in urbanization and longer life expectancy in these countries [8].

### Hypertension in Africa

Traditionally in Africa, communicable diseases and maternal, perinatal and nutritional causes of morbidity and mortality accounted for the greatest burden of morbidity and mortality [9]. This burden is fast shifting towards chronic non- communicable diseases, and by extension CVDs. This phenomenon is what is being termed as a “double burden of disease” [10]. Whereas high blood pressure was almost non-existent in African societies in the first half of the twentieth century, estimates now show that in some settings in Africa more than 40 percent of adults have hypertension [11]. The prevalence of hypertension has increased significantly over the past two to three decades [12]. There were approximately 80 million adults with hypertension in sub-Saharan Africa in 2000 and projections based on current epidemiological data suggest that this figure will rise to 150 million by 2025 [8]. Further, there is evidence that indicates that related complications of hypertension, and in particular stroke and heart failure are also becoming increasingly more common in this region [13, 14]. These trends have been strongly linked with changes in individual and societal lifestyle such as an increase in tobacco use, excessive alcohol consumption, reduced physical activity and adoption of “Western” diets that are high in salt, refined sugar and unhealthy fats and oils.

### Epidemiological Transition

Health and disease patterns change over time in societies depending, among other factors, on the degree of changes in population structure and the rate of economic development, to result in the so-called epidemiological transition [15]. As societies develop, although communicable diseases such as tuberculosis prevail, non-communicable diseases become more prevalent, particularly in urban populations. This is a result of changes in environmental and behavioral determinants such as increasing tobacco use, increasing fat and calorie consumption, and decreasing physical activity and longer periods of exposure to these determinants because of longer life expectancy. Whereas European and North American populations experienced similar changes in demography, determinants, and disease rates over the course of a century, African populations are passing through similar transitions in just a few decades [16]. Traditionally, Africa has been the last major region in the world where the burden of infectious disease still outweighs the burden of non-communicable diseases and injuries [9]. While the rates of decline in fertility and mortality vary considerably across the region, at least one clear pattern is emerging that holds across most of Africa: a steady rise in non-communicable diseases (including cardio-metabolic and respiratory conditions as well as cancers) in the presence of significant, long-standing infectious disease prevalence. As in other parts of the world, the prevalence of hypertension in the sub-Saharan Africa region has increased as a manifestation of the epidemiological transition [17]. Hypertension has become a significant problem in many African countries experiencing the epidemiological transition from communicable to non-communicable diseases [18, 19]. Rural-to-urban migration coupled with acculturation and modernization high blood pressure as observed in Kenyan and Ghanaian epidemiologic studies [20, 21].

### Urbanization in Africa

For the first time, in 2009, Africa's total population exceeded one billion, of which 395 million, almost 40 percent lived in urban areas [22]. This urban population is projected to grow to one billion in 2040, and to 1.23 billion in 2050, by which time 60 percent of all Africans will be living in urban centers. In 2015, Lagos will be the largest urban center in the region with 12.4 million inhabitants. By 2020, Kinshasa's 12.7 million will have overtaken Cairo's then 12.5 million inhabitants. Luanda has recently surpassed Alexandria and is now Africa's fourth largest urban cluster. It is projected to grow to more than 8 million by 2040.

This increasing urbanization is one of the mean reasons for the rise of prevalence in hypertension [23]. The levels of hypertension are structurally higher in urban than in rural settings [20, 24] mainly because of contextual and behavioral factors associated with urban environments such as dietary changes and sedentary lifestyle that together form a complex system conducive for developing hypertension [25]. As the region becomes more urbanized, so will the prevalence of hypertension.

### Population Aging

The world is increasingly becoming older. In less than twenty years there will be globally more people aged older than 60 than children under 10 [26]. It is important to realize that 73% of this older people will live in LMICs. Africa has also remarkable projections in this field. Despite remaining younger than all other continents, Africa will see 13-fold growth in the size of its older population — from 56-million today to 716-million by the end of the century [26]. This growth will outstrip that of any other world region or any other age group. This enormous shift of demographics will have a strong effect on public health in Africa. This increase of older people will lead to growing prevalence of chronic diseases like cardiovascular diseases. As aging is a risk factor for hypertension the prevalence of high blood pressure will be further pushed up in the coming decades because of this demographic shift.

## Prevalence of hypertension in various settings in Africa

Hypertension prevalence data are crucial for understanding the magnitude of the problem, identifying groups at high risk for CVD, and evaluating the effects of interventions in policy and practice [27]. An increase in hypertension prevalence will invariably lead to dramatic rises in the incidence of CVDs and their consequences, which has the potential to overwhelm health care systems [28, 29]. It will also have financial implications for national and local treatment plans because there is increasing evidence that the majority of patients with hypertension will require two or more drugs to achieve blood pressure control [30].

Available data on hypertension prevalence are from a wide range of studies [11, 31, 32], and this limits the opportunity for reliable comparison between different settings. There is heterogeneity in the studies including differences in sampling methods and study settings (predominance of health facility- based studies), the study population, measurement of blood pressure and definition of hypertension as well as the study time periods. Reliable, large-scale, population based data on hypertension in Africa are scarce [33]. In this section we describe the prevalence of hypertension and the determinants for high blood pressure in various settings in Africa garnered from the available published studies and from some nationally representative surveys.

**Figure 1:**
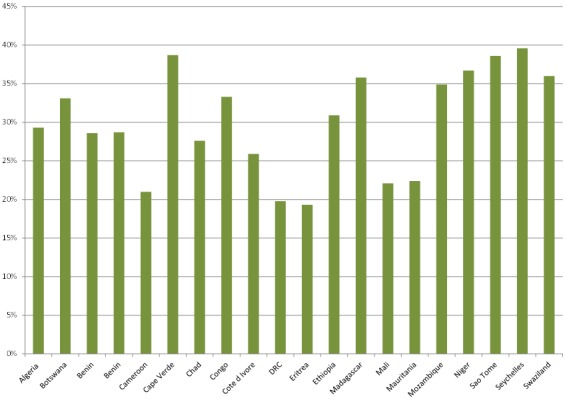
Prevalence of hypertension by sex in selected African countries that participated in the WHO-STEPS surveys (2003 to 2009)

**Figure 2:**
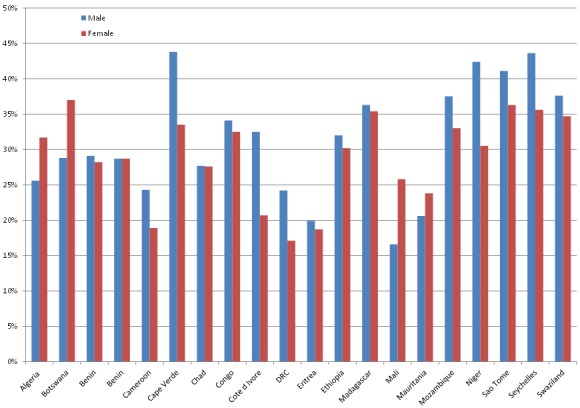
Prevalence of hypertension in selected African countries that participated in the WHO-STEPS surveys (2003 to 2009)

The WHO STEPS survey conducted between 2003 and 2009 in 20 African countries reported high rates of hypertension in most countries, particularly among men (Figure 1). The prevalence ranges from 19.3% in Eritrea to 39.6% in Seychelles (32). The prevalence is for the adult population aged 18 years and above. For more country specific estimates see Figure 2. The prevalence is for the adult population aged 18 years and above. In Africa, hypertension is usually more pronounced in males than in females (11). However, in a few countries there were higher levels of prevalence in women than men such as in Algeria 31.6 percent vs. 25.7 percent in 2003, Botswana 37.0 percent vs. 28.8 percent in 2006 and Mali 25.8 percent vs. 16.6 percent in 2007, for women and men, respectively (Figure 1).

**Figure 3:**
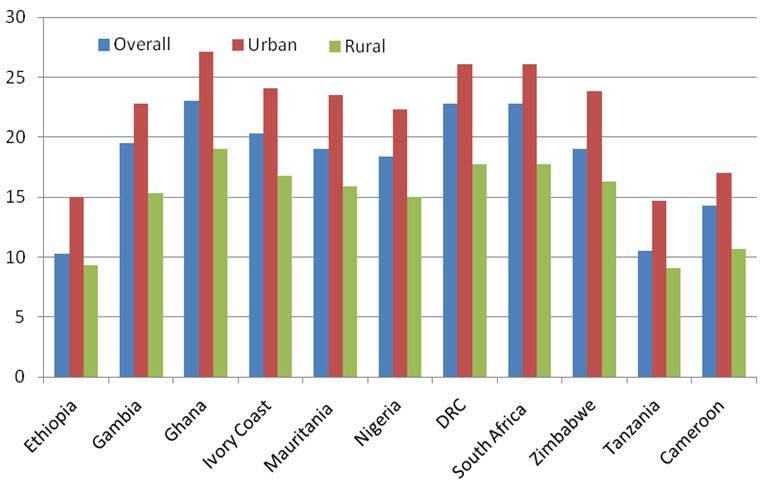
Prevalence of hypertension by rural-urban residence in selected African countries participated in the World Health Survey (2003)

Apart from sex differences in the prevalence of hypertension, there are also large differences based on residence. In all countries where data are available from the World Health Study (WHS), the urban population has a higher prevalence of hypertension than the rural population (Figure 3). We used data from the WHS because those from the STEPS surveys are not disaggregated by area of residence.

In South Africa and Democratic Republic Congo, the urban has almost 10 percentage points higher prevalence than the rural population. This is in comparison to countries like Ethiopia and Tanzania where the prevalence is only a bit more than 5 percent higher. It is noteworthy that since countries are at different stages of the epidemiological transitions, there are some rural populations in some countries whose prevalence is higher than some urban populations in other countries. For instance, rural populations in Ghana, South Africa, and DRC have a higher prevalence than the urban populations in Ethiopia and Tanzania.

**Figure 4:**
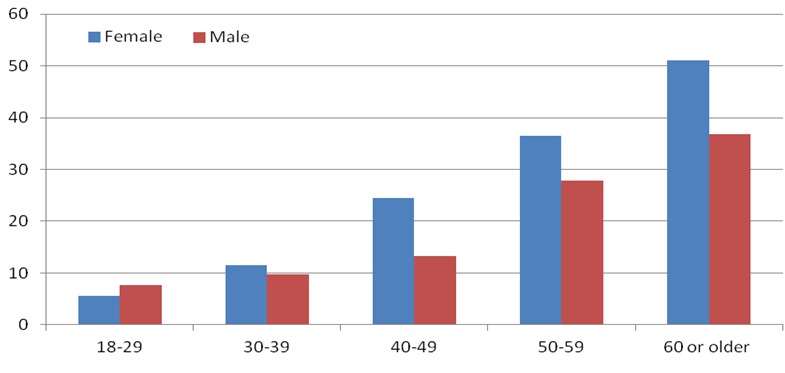
Prevalence of hypertension by age and sex in an urban poor population in Kenya

It is well known that urban averages mask great intra-urban disparities largely due to the presence of large populations in poor slum settlements that characterize most urban centers in Africa. Data from Nairobi collected from the adult population in two slum settlements [34] show a high prevalence of hypertension (overall of 19%) with large sex and age-specific differences (Figure 4). The data also dispel the notion that hypertension is a disease of affluence since the majority of residents surveyed are poor. The data also show a reversal of the general trend of males having higher prevalence than females shown in Figure 1. There is no comparable data from the more

## Risk factors for hypertension in Africa

Hypertension is mainly associated with environmental and lifestyle factors rather than with genetics and has a stronger association and causal link with five particular behaviors: tobacco use, excessive use of alcohol, physical inactivity, unhealthy diet (high salt intake and, insufficient fruit and vegetable consumption) and obesity. Risk factors leading to hypertension can be reversible (modifiable), irreversible (non- modifiable), or associated with other predisposing disorders (Annex 1).

This section describes the distributions of these main modifiable risk factors for hypertension in Africa. Analysis is based on findings in African countries from the 2003 World Health Survey (WHS) and the WHO STEPS survey (2003-2009) on chronic disease risk factors. Data for the described modifiable risk factors are presented at the end of this section.

### Modifiable Risk Factors for Hypertension

**Tobacco Use** Tobacco smoking is known to increase the risk of developing hypertension and cardiovascular diseases like stroke, thrombosis and heart attack. Smoking causes an immediate increase in blood pressure resulting in higher ambulatory blood pressure levels for smokers than for non-smokers. Smoking cessation is known to reduce the overall risk of cardiovascular diseases [35]. In order to reduce smoking at the population level, it is important to implement multi-sectoral interventions like increasing taxes on tobacco products, banning of tobacco advertisements and banning smoking in public spaces [36].

The prevalence of tobacco smoking varied widely in the 2003 WHS. In all countries, men smoked more than women, with the largest disparities observed in Central and Western Africa. Among men, the prevalence of smoking was highest in Southern Africa followed by Eastern Africa. Remarkably, smoking was more prevalent in rural than in urban areas in most countries. Only in Kenya, Mauritania, Senegal and South Africa, were smoking levels higher among the urban population than their rural counterparts. A similar trend is observed in results from the STEPS survey. Daily smokers represented a greater proportion of actual smokers and were disproportionately male. Smoking was more likely in the island nations of Seychelles (22.2 percent), Madagascar (19.6 percent) and Mauritania (19.0 percent) and less pronounced in Western and Eastern African countries. For example, Ethiopia reported prevalence at 4.6 percent in 2006 and Benin was slightly lower at 3.8 percent in 2007.

### Alcohol Consumption

Alcohol consumption is relatively frequent in Africa [37]. There is a direct effect between high levels and specific patterns of alcohol consumption (such as binge drinking) and rising risk of hypertension. The influence of heavy drinking, on increasing blood pressure levels has been described in Nigeria [38]. Interventions to limit alcohol use should be introduced in a multi-sectoral manner and adapted to the local situation. Such interventions, like in reducing tobacco use, include increasing taxes on alcohol, and banning alcohol advertising especially to young people [36].

In both the STEPS and the World Heath Survey (WHS), prevalence of alcohol consumption did not vary significantly between rural and urban populations. This implies an increase in prevalence rates in the rural, to levels similar to those previously only found in urban areas. Men show higher prevalence of alcohol consumption in terms of current and daily drinkers and are more likely to be frequent drinkers (defined as having more than four standard drinks/day on average on most days of the week) than women.

### Inadequate Physical Activity

Adequate physical activity has been shown to have many health-promoting effects and has a direct, independent role in reducing hypertension [39, 40]. Traditionally, it has been thought that a high level of physical activity could in part explain the low levels of chronic diseases found in most of Africa. However, the amounts of physical activity have been decreasing as a result of the high rate of urbanization that has been occurring across the continent [41]. Few studies on the physical activity patterns of African populations have been published.

From the STEPS survey, adequate physical activity, defined as more than 150 minutes per week walking/moderate activity/vigorous activity, reported by males was markedly higher than that of females. In the WHS, women also reported lower levels of physical activity than men, probably because of their traditionally defined roles, such as caregiving, that require less physical strain. Adequate physical activity is more prevalent in rural than urban regions of Africa, which partly explains the high prevalence of obesity in urban areas.

### High Salt Intake

A high intake of sodium is common, in Africa mostly from salt used to preserve food or to make it tastier [42]. Also, salt is added to already-prepared food by the consumer, as processed food is rare. Decreased salt intake not only reduces blood pressure and related CVD risk, but has other beneficial cardiovascular effects that are independent of and additive to its effect on blood pressure [43]. It has been reported to have a direct effect on reducing stroke, left ventricular hypertrophy, aortic stiffness, and chronic kidney disease and proteinuria [36]. For that reason, it is reasonable to infer that the total impact of reducing salt intake on cardiovascular outcomes could be greater than those expected from blood pressure reduction only.

Few intervention studies have been conducted to show that a reduction in salt intake and an increase in potassium improve the blood pressure in African populations. A study done in Tanzania indicated that a low sodium diet leading to a low urinary excretion level of 52 mmols per day, reduced blood pressure in normotensive people significantly within four to five days [44]. A study in Kenya reported that supplementation with potassium in newly diagnosed patients with hypertension reduced the blood pressure to a level similar to that found in patients treated with a diuretic [45]. These studies provide evidence of the impact of community-based and context specific salt-reduction programs in Africa where most salt is still discretionary rather than from processed foods as is the case in developed countries.

### Insufficient Fruit and Vegetable Consumption

Fruit and vegetable consumption is one element of a healthy diet and varies considerably among countries, reflecting economic, cultural and agricultural production environments [46]. Most of the benefits of fruits and vegetables come from reduction in CVD and risk factors, particularly hypertension. In addition to a high salt intake, many people in Africa often eat insufficient fruits and vegetables, resulting in low potassium intake. This in turn is associated with higher blood pressure in some patients; a potassium intake of 90 mmol/day is recommended [47].

The STEPS surveys reported sufficient fruits and vegetables intake, defined as five or more servings of fruits or vegetables per one typical day, as being very low across all countries. There were slight gender variations in mean servings of fruit and vegetables reported with women more likely to consume the recommended number of servings than men. This finding was mirrored in the WHS where also, rural people were more likely to consume insufficient amounts of fruits and vegetables compared to their urban counterparts.

### Obesity

The World Health Organization (WHO) defines obesity as a condition in which excess body fat has accumulated to such an extent that health may be adversely affected. The degree of body weight is usually expressed as BMI; this is the ratio of weight in kilograms to the square of height in meters. The BMI is used to classify a person's body weight as underweight (BMI less than 18.5), normal weight (BMI 18.5-24.9), overweight (BMI 25-29.9), or obese (BMI greater than 30). Obesity greatly increases the risk for hypertension and has also been shown to be associated with coronary artery disease and some cancers, and to reduce life expectancy [48]. As obesity is rapidly rising in different countries, it will be important to share best practices to reduce this trend [41].

STEPS findings show that there is a high prevalence of overweight and obesity in many African countries particularly among urban women. The mean BMI was significantly higher for women compared to men across all countries as was the standardized prevalence of overweight (BMI> 25Kg/m2) and obesity (BMI>30Kg/m2). Presumably, the lower male prevalence relates, in part, to the much higher rate of heavy manual labor commonly reported in men while the higher prevalence rates among women may be explained by the physiological changes related to pregnancy and childbirth, and cultures that put a high premium on overweight and obesity among women. In Table 1, we show the prevalence of five key risk factors for hypertension in selected countries based on data from the nationally representative STEPS surveys.

**Table 1:** Prevalence of Risk Factors for Hypertension in Selected Countries from the WHO STEPwise Surveys (2003-2009)

Country	Tobacco smoking*	Alcohol intake**	Inadequate physical activity	Insufficient fruit/vegetable intake	Obesity
Algeria (2003)	15.1	-	37.1	87.1	16.6
Cameroon (2003)	8.2	-	26.6	-	22.3
Congo (2004)	11.1	-	-	-	8.6
Seychelles (2004)	22.2	87.3	42.2	78.8	25.1
Eritrea (2004)	8.7	28.3	33.7	97.6	4.0
Côte d'Ivoire (2005)	14.4	34.0	24.3	83.5	8.5
DRC(2005)	7.6	-	23.1	86.5	8.2
Madagascar (2005)	19.6	31.7	46.5	72.6	2.2
Mozambique (2005)	18.7	45.2	85.3	95.0	7.5
Ethiopia (2006)	4.6	45.7	42.1	98.9	7.1
Mauritania (2006)	19.0	-	10.3	94.3	24.7
Botswana (2006)	19.7	18.7	43.4	96.6	15.6
Benin (2007)	3.8	36.9	66.2	94.7	21.6
Cape Verde (2007)	9.9	40.3	60.3	86.1	10.5
Mali (2007)	15.8	3.8	23.4	80.2	17.9
Niger (2007)	5.0	0.2	56.1	96.3	3.2
Swaziland (2007)	7.1	11.8	53.3	87.4	24.3
Benin (2008)	8.8	48.8	80.8	78.5	9.4
Chad (2008)	11.2	17.0	41.1	84.8	13.7
Sao Tome (2009)	5.5	84.5	65.9	83.3	11.7

* Current smoker

** Current user i.e. has consumed an alcoholic drink in the last seven days before the survey

## Levels of awareness, treatment and control of hypertension in Africa

The low rates of awareness, treatment and control of hypertension in Africa are a major public health concern as the population in this region is growing [49]. The low levels of all these indicators imply that there will be significantly large populations of hypertensive patients unaware of their increased risk of hypertension-related complications in the coming years. Studies in Africa have shown that many people with hypertension are unaware of their condition, many of those who are aware are not on treatment, and many of those treated are not well controlled [50]. A possible contributory factor would be the affordability of the cost of health care, which remains a major barrier in the African setting as out-of-pocket spending is the main source of funding for health care costs [51].

Another common occurrence is the non-adherence to treatment and follow-up for hypertension. In one intervention study in Cameroon, for instance, just about half of the participants were still in the program at one-year follow-up [52]. Indeed, patients are expected to be treated and controlled only if they can access appropriate health services, receive adequate advice and prescriptions and subsequently afford and adhere to those prescriptions. Increasing awareness, treatment and control rates of hypertension will have a huge impact on CVD prevention in Africa [53]. For instance, data from the national health and nutrition examination survey (NHANES) showed that the age-adjusted percentage of adults with hypertension whose blood pressure was controlled increased from 48.4% in 2007-2008 to 53.3% in 2009-2010, whereas in Africa only 5 to 10 percent is controlled at a blood pressure of less than 140/90 mmHg [24, 54, 55].

**Figure 5:**
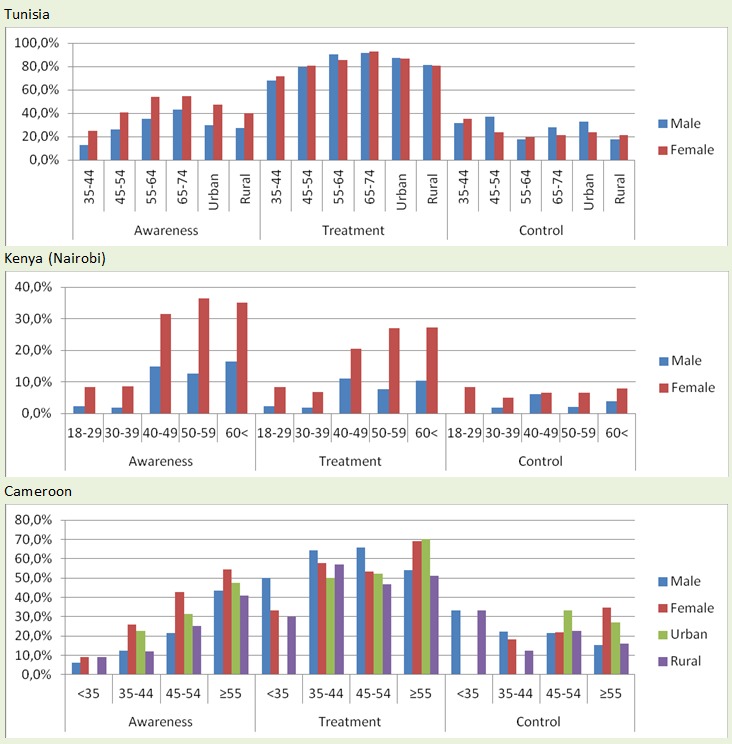
Rates of hypertension awareness, treatment and control in selected african countries by sex

Studies in Tunisia, Cameroon and Kenya assessed the levels of awareness, treatment, and control (Figure 5). All levels of awareness, treatment and control were found to be significantly lower for men than women, lower in rural than urban areas and to be higher among older age groups (Figure 5).

## Care and management of hypertension

Management of hypertension involves lifestyle changes as well as drug treatment. Lifestyle measures are useful both in the control of high blood pressure and in risk factor management. They include weight reduction, increase in physical activity, reduction in salt intake, moderation of alcohol intake and cessation of smoking. As regards drug treatment, several classes of drugs are recommended for the treatment of hypertension and this is dependent on co-existing disease conditions and on the presence or absence of complications. Often, more than one drug is necessary to achieve control. The five main classes with proven effect are: Beta-blockers (BB), Diuretics (DIU), Calcium channel blockers (CCB), Angiotensin converting enzyme inhibitors (ACEI) and, Angiotensin receptor blockers (ARB).

In Africa, providing medication is considered an important and cost effective way to reduce hypertension [56], but accessibility to and cost of the treatment are very often forgotten. Currently, African countries are 80 percent below the global average for pharmacological spending and 20 percent below the global average of behavioral risk factors for hypertension [57]. There is a lot of opportunity for hypertension control through improving availability of medication as has been widely reported in the literature.

The efficacy of blood pressure lowering medications is well demonstrated and treatment of high-risk individuals has been advocated as a major strategy for CVD prevention in all regions, including Africa [4]. However, managing hypertension -or elevated total CVD risk- is challenging in Africa for a variety of reasons, including lack of availability of drugs, high treatment costs, as well as inadequacy of health services for identification and management of CVD and its risk factors [58]. Moreover, health systems in most LMICs are already stretched by the high burden of infectious diseases such as HIV, TB and malaria. Furthermore, individuals who struggle with a broad range of day-to-day problems may discount the benefit of long-term treatment for silent and painless conditions that do not immediately jeopardize their health. There is also evidence that Africa is undergoing very rapid transitions characterized by increasing urbanization, including the adoption of unhealthy lifestyles. These transitions are paralleled by changes in the profile of chronic disease risk factors including blood pressure.

Recently, there has been increasing awareness of the threat that the looming non-communicable diseases epidemic poses to the health of the population in the region at different levels. This increasing awareness has catalyzed initiatives aimed at improving access to detection and care of chronic diseases including hypertension in many countries of the region. The pace of the above developments suggests that data on the burden of hypertension must be updated regularly, in order to provide the reliability needed in drawing up effective health service and policy solutions.

Historically, hypertension guidelines focused on blood pressure values as the only or main variables determining the need and the type of treatment. The current approach emphasizes that diagnosis and management of hypertension should be related to quantification of total (or global) CVD risk. Table 2 shows the recommended therapeutic approach based on CVD risk stratification.

**Table 2:** Therapeutic Guidelines for Managing Hypertension in adults [Adapted from 2007 ESC/ESH Recommendations]

Other risk factors, sub-clinical organ damage or disease	Blood pressure levels (grades according to the ESC classification) in mmHg				
	SBP 120-129 or DBP 80-84 (Normal)	SBP 130-139 or DBP 85-89 (High normal)	SBP 140-159 or DBP 90-99 (Grade 1 HT)	SBP 160-179 or DBP 100-109 (Grade 2 HT)	SBP ≥180 or DBP ≥110 (Grade 3 HT)
No other risk factors	No blood pressure control interventions	No blood pressure control intervention	Lifestyle changes (several months) then drug treatment if blood pressure is uncontrolled	Lifestyle changes (several months) then drug treatment if blood pressure is uncontrolled	Lifestyle changes + immediate drug treatment
One-two risk factors	Lifestyle changes	Lifestyle changes	Lifestyle changes (several months) then drug treatment if blood pressure is uncontrolled	Lifestyle changes (several months) then drug treatment if blood pressure is uncontrolled	Lifestyle changes + immediate drug treatment
Three or more risk factors, metabolic syndrome, sub-clinical organ damage or diabetes	Lifestyle changes	Lifestyle changes+ consider drug treatment	Lifestyle changes + drug treatment	Lifestyle changes + drug treatment	Lifestyle changes + immediate drug treatment
Diabetes	Lifestyle changes	Lifestyle changes + drug treatment			
Established cardiovascular or renal disease	Lifestyle changes + immediate drug treatment	Lifestyle changes + immediate drug treatment	Lifestyle changes + immediate drug treatment	Lifestyle changes + immediate drug treatment	Lifestyle changes + immediate drug treatment

**Notes:** SBP: systolic blood pressure; DBP: diastolic blood pressure; HT: hypertension.

### The Use of Hypertension Management Guidelines

Few guidelines for the management of hypertension in Africa have been published. The region as a whole published its most recent guidelines in 2003 [59], and these share many similarities to the WHO guidelines [60]. These guidelines in particular focus on an arbitrary cut-off value above which certain interventions, including pharmacological therapy, should be initiated. One problem with this approach is that the arbitrary cut-off point continues to change. Blood pressure is associated with a continuous and graded risk of stroke and ischemic heart disease over a long range down to at least 115 mmHg. Since there appears to be no clear cut-off at which the risk is zero above 115 mm Hg, then any level above it is arbitrary. Indeed, most of the disease burden resulting from blood pressure, lipids, and excess weight occurs in the large majority of the population with non-optimal levels but without hypertension per se as defined by the arbitrary cut-offs in multiple guidelines. Furthermore, the choice of a cut-off has real policy and cost implications..

In Africa, if the definition for treatment eligibility were 160/95 mm Hg, which was the cut-off for guidelines in South Africa in 1995, then approximately 4 percent of the adults over the age of 30 would be eligible for treatment. Under the current WHO-ISH guidelines, which use 140/90 mmHg as a cut-off, nearly 22 percent of the population is eligible for treatment, which is nearly a six-fold increase. If the cut-off were eventually dropped to 120/80 mmHg, which the United States Joint National Commission on Hypertension now defines as pre-hypertension, then nearly 70 percent of the adult population in South Africa would be eligible for intervention. Even if the interventions do not include drug treatment, these are large increases in the proportion of the population that would require lifestyle interventions and follow-up, which could not be sustained given the current human and financial resources devoted to healthcare in Africa.

There will be two major challenges, however, for those implementing risk-based guidelines in African countries (i) to build the capacity of current physicians or other cadre of health professionals who, over the years have been trained to target individual risk factors, and (ii) to find simplified measures of assessing risk, given the high cost and impracticality of measuring cholesterol in many regions of Africa. The cost-effective use of health services to control hypertension is particularly needed in countries with multiple burdens of disease and these countries will have to set priorities appropriately in order to use scarce resources most effectively. The failure of countries to adopt the necessary steps, early in the twenty first century, to promote healthy lifestyles, however difficult such decisions might be, will inevitably lead to increasing levels of hypertension and CVD in the populations of African countries. This, in turn, will be the cause of an avoidable chronic disease epidemic within the next few decades.

The crucial role of health research in general, and cardiovascular disorders research in particular cannot be overemphasized. Research is essential in formulating a rational health care policy, evaluating the performance of CVD control interventions and making managerial decisions in the health sector [61]. The need for training and funding for research, and especially the leadership required to develop and sustain research activity, will require a multidisciplinary, multidirectional, collaborative approach at national and international levels, as well as firm commitment from African governments [29].

## Economic cost of hypertension in Africa

The economic burden of CVD in Africa is significant [62]. CVDs will cost the continent billions of dollars in the next decade [63]. Hypertension remains the number one cause of significant financial burden, including the cost of caring for all the complications arising from it like stroke, ischemic heart disease and congestive heart failure [64]. The financial burden comes in the form of direct healthcare costs related to treatment of CVD and its risk factors. These costs are borne by the individuals, governments, and the private sector.

Furthermore, there are numerous indirect costs related to hypertension, data for which are, fragmented for most African countries. These costs include the lost productivity of workers struck by stroke, heart failure, and ischemic heart disease [62]. Other costs include the lost savings and assets that are foregone when families must meet catastrophic healthcare expenditures such as those associated with rehabilitation following stroke or dialysis following renal failure. Added to that are the major economic and social (opportunity) costs to families who - in the near absence of formal care systems - need to provide often intensive long term care to older relatives. In spite of the current relatively low prevalence of hypertension in some countries, the total number of people with hypertension in LMICs is high and a cost analysis of possible anti-hypertensive drug treatment indicates that LMICs cannot afford the same treatment as in high income countries. This is because African countries have limited resources to devote to hypertension in light of other competing health priorities.

The average amount of healthcare expenditure as a percentage of gross domestic product (GDP) for African countries is 6.3 percent. There is however a wide range of this metric ranging from 2.5 percent in the Democratic Republic of Congo to 12.9 percent of GDP in Malawi. There is also a wide range of health care expenditure per capita across African countries from as little as $6 per capita in Ethiopia to as much as $390 per capita in South Africa. Nonetheless, the amounts are still quite small compared with $ 3727 per capita for high-income countries. Effective management of hypertension usually requires treatments with more than one drug. A study in Ghana, found that only 18 percent of a group of patients with hypertension had one drug prescribed, whereas 60 percent had two drugs and 22 percent had three or more drugs prescribed. The use of two or more drugs will inevitably result in a high cost for anti-hypertensive medication, especially when newer medicines are used. However, there are cheaper, older, and effective medications available in most African countries. “Number needed to treat” analyses showed that the cost of drugs to prevent one death is US$ 14,000 to US$ 1 million in the United States, depending on which drug used [65]. Obviously many African countries cannot afford such high costs of treatment for one condition with many other competing health priorities and a limited resource envelop.

## Hypertension prevention and control initiatives

Hypertension is one of the most important modifiable risk factor for CVD, yet the control of this condition in Africa is far from adequate. Available data from a few countries in Africa have highlighted the increasing importance of CVD and other chronic non-communicable diseases in this region, and some of these countries have taken steps to develop relevant policies and programs to address this issue [66]. For a long time, the priority in most African countries has been the prevention and control of communicable diseases. Recently, however, the attention has begun to shift to the control and prevention of non-communicable diseases, including hypertension, in view of the rising burden of non-communicable diseases. For this, an integrated approach to the prevention and management of non-communicable diseases, irrespective of cause, is needed in primary health care (PHC) settings [27]. It is likely that hypertension is particularly poorly detected and treated in PHC settings in many African countries given the low levels of awareness, treatment and control as described above. Countries in the region should therefore be encouraged to establish country-specific recommendations for the prevention and management of hypertension, as already recommended by the World Health Assembly (WHA) and the WHO Regional Committee for Africa. Chronic disease interventions selected for use in PHC must lead to productive changes in risk status and outcomes, be cost effective, and be financially and logistically feasible, to be available for implementation across a range of resource settings.

### Strategies and interventions to prevent/control hypertension

**Figure 6:**
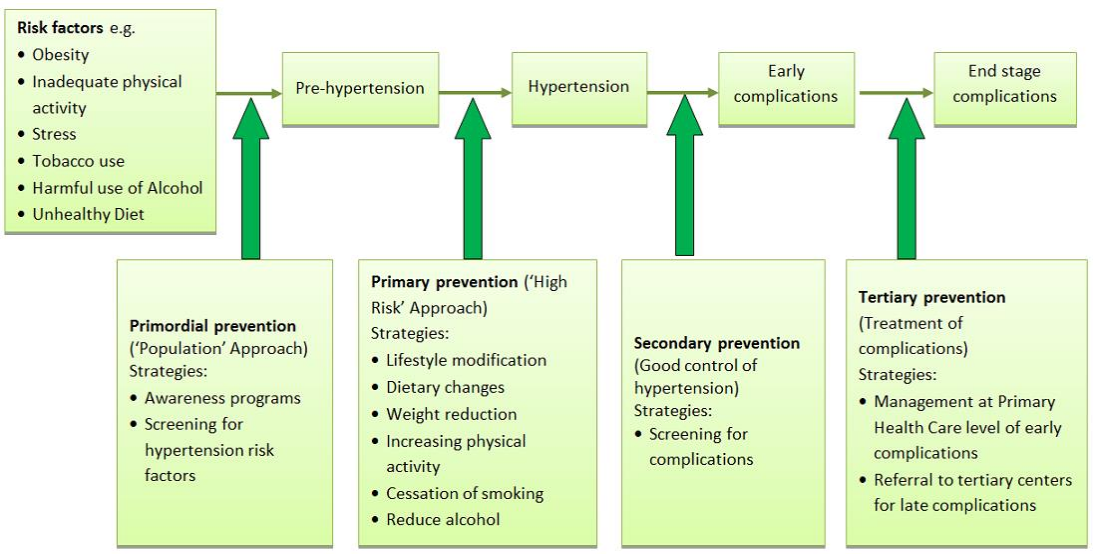
Strategies for the prevention and control of hypertension

Within the context of limited resources, in most of Africa, the greatest gains in controlling the hypertension epidemic lie in its prevention, or at least early detection and adequate control. For most LMICs the major obstacle to the control of blood pressure is the absence of appropriate services at the primary health care levels of the health service delivery system [36]. The commonality of many risk factors for hypertension and other CVD justifies an integrated approach to prevention and control. This requires tackling at several levels (a) Primordial prevention i.e. the reduction of the risk factors of hypertension in the general population and thereby decreasing the risk of developing hypertension in future (b) Primary prevention i.e. prevention of the condition in those who have prehypertension (c) Secondary prevention i.e. prevention of complications in those who have already developed hypertension, and (d) Tertiary Prevention i.e. preventing progression to end stage complications in those who have already developed some associated complications (Figure 6).

### Primordial prevention

Primordial prevention depends on health policies that create a congenial environment that promotes healthy behaviors. It entails population-wide education programs, which in turn depend on many factors, including political commitment, advocacy by health professionals and involvement of community leaders and the mass media [67]. The challenge is to urgently and adequately raise awareness in the community at large and generate action. Effective population control of hypertension demands an improvement in awareness about hypertension and its role in CVD risk (among both health professionals and the general population), a systematic assessment of overall CVD risk among people who come into contact with health services and an increase in the effectiveness of non- pharmacological and pharmacological interventions [50]. Another important measure to promote is the regular checking of blood pressure for all adults (including young ones). In addition to prevention, awareness when one has hypertension is important. Efforts should be made to detect hypertensive patients early before irreversible organ damage occurs, and to provide them with the best possible and affordable non-pharmacological and pharmacological treatment based on current management recommendations.

Population screening activities are an important component of any prevention or control program and are particularly important in populations at high risk for developing hypertension, for example, urban populations and those with limited access to medical care [50]. However, screening has to be linked with proper diagnosis (e.g. repeated blood pressure measurements), assessment of overall cardiovascular risk and therapeutic follow-up [27]. To be sustainable, a typical screening program needs to be supported by (i) health education programs that suit local conditions and socio-cultural realities; (ii) awareness- raising programs that target patients and the general population through media and other local communication channels, and; (iii) dissemination of context specific recommendations for management and assessment of high blood pressure and CVD risk factors; and (iv) inter-regional and global CVD information exchange networks.

### Primary prevention

The next step after the high risk group is detected is to give advice on lifestyle modification based on the CVD risk. The good news is that many risk factors of hypertension can be modified. In Africa, one hypothesis is that the rural diet is relatively protective, but is abandoned with urban exposure, with less carbohydrate and higher fat intake. Sodium restriction is feasible as a solitary measure, but to achieve general application, it clearly requires national governments’ commitment and multiple messages from different sources, as well as salt-reduction interventions by the food industry [42]. The major problem is how to get the lifestyle messages across and how to implement them. The high risk group can be empowered through health education on lifestyle modification (LSM) and their benefits, with the help of health workers at primary care levels.

### Secondary prevention

While all efforts must be taken to prevent hypertension, there is also a clear need for appropriate care for those who already have the condition and its related complications. In general, the first aim is to cast the net widely and to make antihypertensive medication available to as many people with hypertension as possible. With regard to low-cost therapy, the first crucial point is which cutoff blood pressure values to use. Using 160 mm Hg systolic as a cutoff point would mean that only nine people would have to be treated each year to prevent a cardiovascular event and 50 would have to be treated each year to prevent one death [67]. Lower cutoff points, such as 140 mm Hg systolic, now regarded as ideal when economically feasible, lead to greater numbers needed to treat to prevent either a cardiovascular event or CVD-related death. Thus, the higher cutoff levels, the more practical though less medically desirable is the policy for resource-constrained African countries. The way forward out of this dilemma could be greater use of global CVD risk calculations for the individual hypertensive, which is a more cost-effective approach than decisions based on just the cutoff blood pressure level [68].

### Tertiary prevention

Only five percent of individuals with hypertension will ultimately need tertiary care facilities for management of their advanced stage of hypertension complications. Examples of tertiary care needed include dialysis or kidney transplantation for renal failure, coronary angiography or bypass/angioplasty for coronary artery disease. Effective tertiary centers are costly and there is need for efficiency, quality control and accountability. Medical insurance schemes are one way of making such tertiary care accessible to many people while controlling unnecessary costs and ensuring quality through the schemes’ internal quality assurance mechanisms.

### Action plan for prevention and control of hypertension (Annex 2)

As the interest in CVDs and their risk factors such as hypertension grows among policy makers and government institutions a number of documents have been developed with recommendations on how to tackle this public health concern at different levels. Specifically, the WHO has published several reports with action plans regarding non-communicable diseases, CVD and hypertension. Below is an overview of the various recommendations and guidance documents adapted to the situation of hypertension in Africa.

## Discussion

Africa faces an unprecedented epidemic of CVDs. Hypertension is the key driver of cardiovascular complications. Whereas high blood pressure almost did not exist in native African populations in the first half of the twentieth century, hypertension now affects between 20 percent and 40 percent of these populations. Lowering blood pressure and controlling hypertension is key to CVD prevention.

Many countries in Africa are undergoing a rapid demographic and epidemiologic transition. While much attention in the region has been focused on communicable diseases such as malaria, tuberculosis, and HIV/AIDS, changes in demographic and determinants of health, particularly changes in lifestyle associated with urbanization, have resulted in an epidemiological and nutrition transition towards a greater prevalence of non-communicable diseases. The dual burden of persistent infectious diseases and emerging chronic diseases such as hypertension, poses a serious threat to population health in the region.

Prevalence and incidence of both hypertension and pre-hypertension are high. Efforts to prevent or attenuate high blood pressure could lead to a substantial reduction of complications. Lifestyle modifications play a crucial role in preventing elevation of and better control of high blood pressure. Weight loss, control of sodium intake and diet, and promoting physical activity are essential steps towards this direction. However, when medications are needed to reduce blood pressure levels, the selection of the appropriate drugs is important not only for effective control but also to minimize side effects.

Awareness, treatment, and control of hypertension in Africa are lagging behind many world regions [69]. Significant numbers of individuals with hypertension in Africa are unaware of their condition and, among those with diagnosed hypertension, treatment is frequently inadequate. Detection, prevention, and treatment of hypertension should now be regarded as a high priority in Africa [70]. Establishing factors associated with awareness and management is an essential starting point in preventing an increase in the burden of hypertension- related CVD [50]. While it is true that enormous challenges exist in the control of communicable diseases in Africa, non-communicable diseases such as hypertension are also important threats to the health of the adult population in many countries. Controversy exists, however, over the priority these conditions deserve in the competition for scarce resources. Unfortunately, these discussions take place in an information vacuum, since it is impossible to define the burden of chronic conditions in societies where health statistics are unavailable. The scarce resources available must benefit the whole population. Research into non-communicable diseases, particularly cardiovascular disease in Africa should be seen as vital especially where it can inform resource-allocation decisions.

The problem of defining a strategy for hypertension control confronts most societies [27]. Hypertension is fully treatable, but social and economic conditions in many African countries make the implementation of blood pressure control programs difficult [71]. Lack of a clear strategy based on evidence has undermined these efforts further. Inadequate funds, inexperience, and lack of infrastructure remain important barriers to hypertension detection and treatment [63]. Accordingly, the overall management of hypertension is as much a socioeconomic as it is a therapeutic problem. Screening ideally not only detects hypertension but also forms the basis for education and therapy.

There remains a wide gap between international hypertension guidelines and national hypertension guidelines. For one, only in a handful of countries, for example South Africa, have national hypertension guidelines been developed and launched [59]. Separately, another factor not often included in standard treatment and care models is the indirect cost to the patient on transport and other health care costs. Practice guidelines serve as useful tools for clinical decision making. Countries in Africa should therefore be encouraged to establish country-specific recommendations for the prevention and management of hypertension as already recommended by the World Health Assembly and the WHO Regional Committee for Africa. For primary and secondary CVD risk reduction in hypertensive patients, the evidence-based recommendations for blood pressure treatment, including smoking cessation, and healthy lifestyle behaviors outlined by WHO guidelines should be followed [72]. Measures should be taken to increase the adherence to practice guidelines, and improve both primary care health providers and patients’ knowledge. Evaluating and auditing of adherence to the national guidelines and management in primary healthcare should be done regularly to ensure that appropriate care is provided.

Collating information on risk factors for cardiovascular diseases in Africa is an enormous task that is achievable only through collaboration. An active approach to hypertension must be driven by the ministries of health as well as by local organizations, with support from influential bodies such as the International Forum for Hypertension Control and Prevention in Africa. The current enthusiasm for collaboration is crucial for the development and implementation of health-care policies throughout the region. This collaboration is especially important when attempting to validate any developed guidelines for treatment or prevention of non-communicable diseases in Africa. The epidemiology of hypertension morbidity will at least provide the starting point for better health-care planning, which could mirror the way communicable diseases have been handled.

## The way forward

### Development of national plans of action

An analysis in the different countries should be made of which stakeholders are involved in CVD prevention and control, which activities are currently ongoing. These stakeholders should be given the forum and opportunity to come up with a national plan of action following the WHO recommendations.

### Reduce risk factors through policies

Public health policies should be focused on reducing risk factors for hypertension and CVD in general like tobacco use, excessive alcohol intake, physical inactivity, and unhealthy diets. This can be done by fully implementing already existing policies like the Tobacco Control Act and crafting new policies to address the other risk factors.

### Surveillance and monitoring

National and regional bodies should take the lead in strengthening hypertension surveillance and monitoring efforts. Data are critical for determining the burden of hypertension, characterizing the patterns among sub groups of the population, assessing changes in the problem over time, and evaluating the success of interventions. Effective monitoring and surveillance systems need to be in place, to track progress in reducing the prevalence of hypertension, and increasing awareness, treatment and control of hypertension.

### Improve health systems structure

Besides implementing policies that are covering the society in general there should also be a strong focus on development and improvement of health service delivery system to address the management of chronic NCD. This can be done through community based screening programs and strengthening Primary Health Care settings to manage simple cases as well as establishing strong referral links to ensure continuity of care among diagnosed patients.

### Improve quality of care

It is essential that quality of care for hypertension patients improves as part of efforts to strengthen the health service delivery system for the management of chronic NCDs. This can be done through rigorous training of health care staff like nurses, community health workers and clinical officers. In order to ensure consistent quality of care, national standard guidelines for treatment and management of hypertension should be developed and implemented.

## Annexes

**Annex 1:** Risk factors for hypertension (http://www.panafrica-med-journal.com/content/16/38/full/annex/annex1.pdf)

**Annex 2:** Summary of WHO Action Plans 2008-2012 (84) for Prevention and Control of Hypertension and CVD (http://www.panafrica-med-journal.com/content/16/38/full/annex/annex2.pdf)
